# Mr. Roboto (domo arigato, if you like)

**DOI:** 10.1002/bco2.54

**Published:** 2020-11-04

**Authors:** John W. Davis

The rock band Styx released a song of this name in 1983. I was in ninth grade at the time and played guitar in a band. We sort of laughed at this song—seemed a rather ridiculous departure into 1980s synthesizer‐based rock for a band that was rooted in classic guitar riffs and keyboard ballads in the 1970s. The band went full in with the themes from the lyrics—a full length music video and a linked rock opera story line. Fast forward a few decades, and the song does show up on my play list in the operating room as part of my Styx Greatest Hits album. Often I get a shout out from the room, as if this song is some sort of anthem for a robotic surgeon.

As this issue of *BJUI Compass* features a review article on new robotic surgery platforms, this song has been on my mind. Looking up the lyrics on‐line, the song is really not about robots but rather human emotions and frustrations. Pausing for a brief trip down a rabbit trail, I am able to read these lyrics on‐line, since almost all pop songs now have internet sites posting the lyrics, and discussions on the meaning and backstories. For the musicians interested: sheet music available to download, and in many cases YouTube videos on how to play the instrumental parts. This is such a departure from my brief era as a rock guitarist in the 1980s—we had to record songs on cassette tapes off the radio and play/rewind them endlessly to figure out the lyrics and the instrumental parts. In many cases, you really could not figure out what they were singing (looking right at you, Sting). Follow this link for an amusing list of top 40 “mis‐heard” song lyrics: https://www.nme.com/blogs/nme-blogs/misheard-song-lyrics-6787

Ok—back from the rabbit trail. Let's get to this month in the *BJUI Compass*, with contributions from South Korea, Australia, and Japan.

**FIGURE 1:**
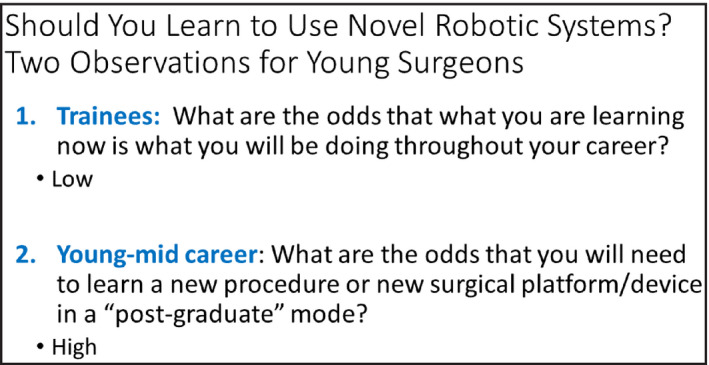
A slide from my lecture on single port robotics—trainees are often less enthusiastic to learn, but the long vision would predict that young surgeons will need to adapt to novel systems several times in a career

**FIGURE 2:**
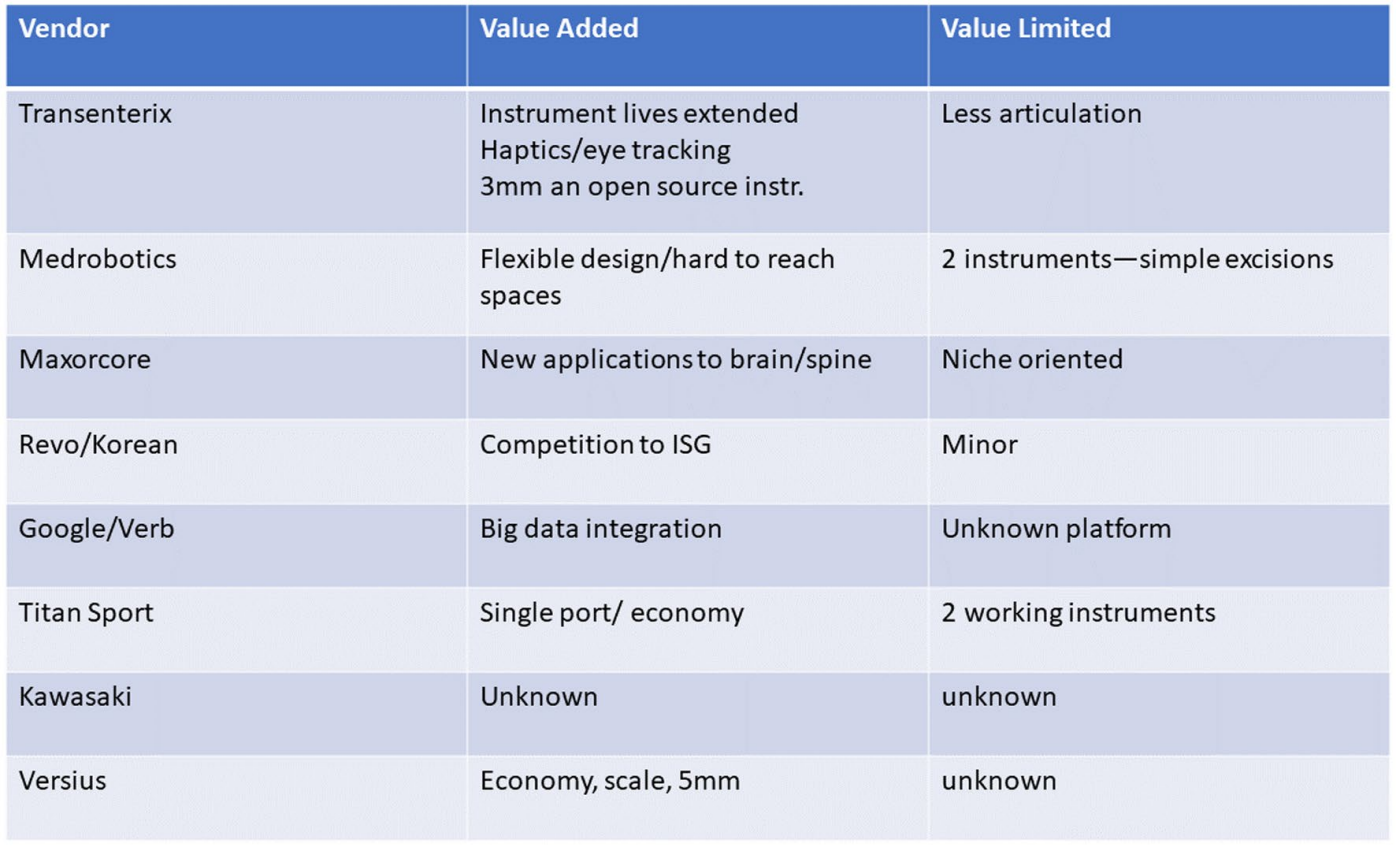
A slide from a 2018 lecture on novel robotic systems delivered at the Société Internationale d’Urologie meeting in Seoul, South Korea. My take on new robotic systems has been to evaluate them by the value added versus the value limited compared to currently available systems. With these eight systems, you can see a variety of areas where they may be improved, be limited, or be too early to know

**FIGURE 3:**
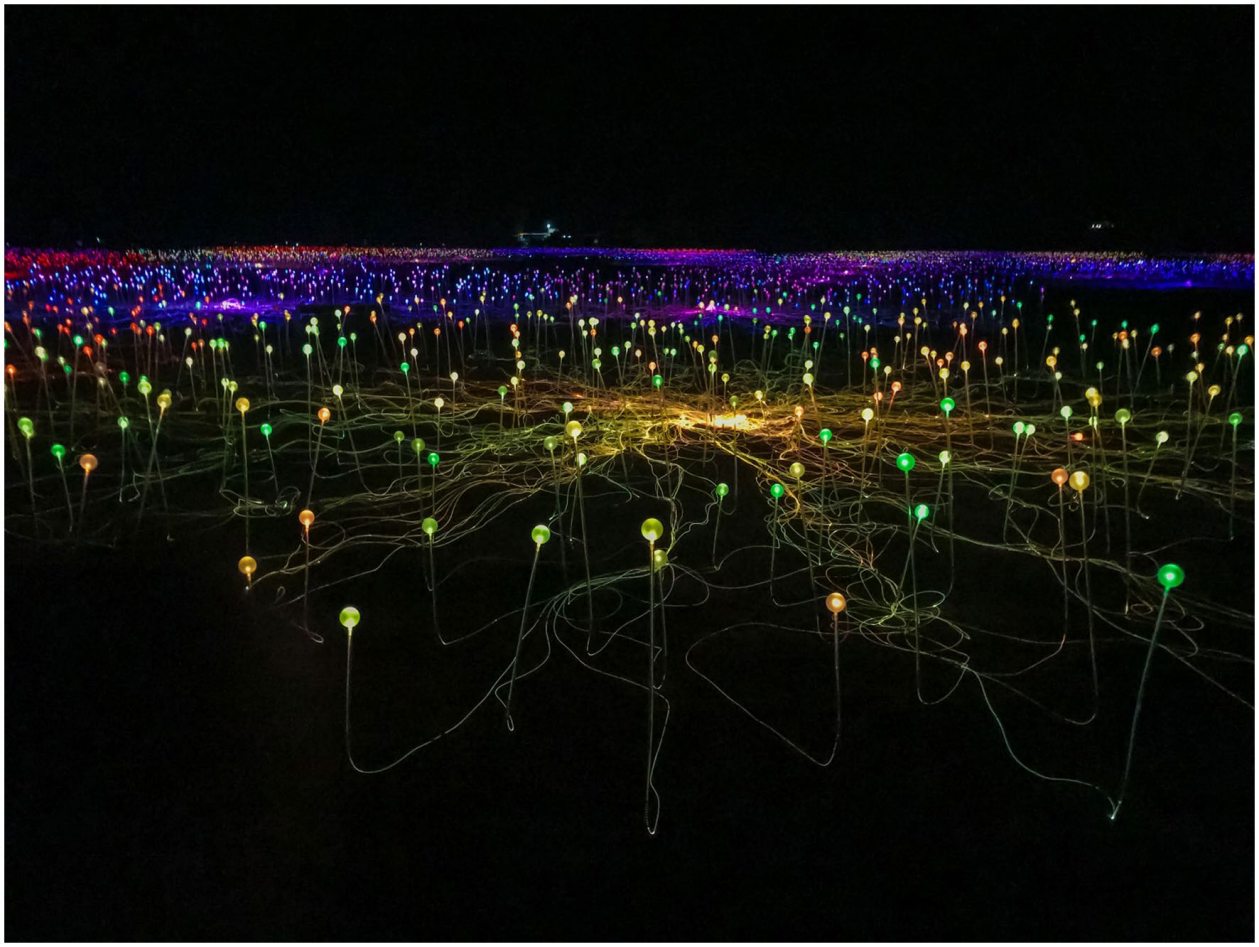
Field of Light at the base of Uluru in the Northern Territory of Australia—a display of more than 50 000 solar powered lights in the vast desert of the Outback. The connections feel like a vast neural network covering the size of four football fields

**FIGURE 4:**
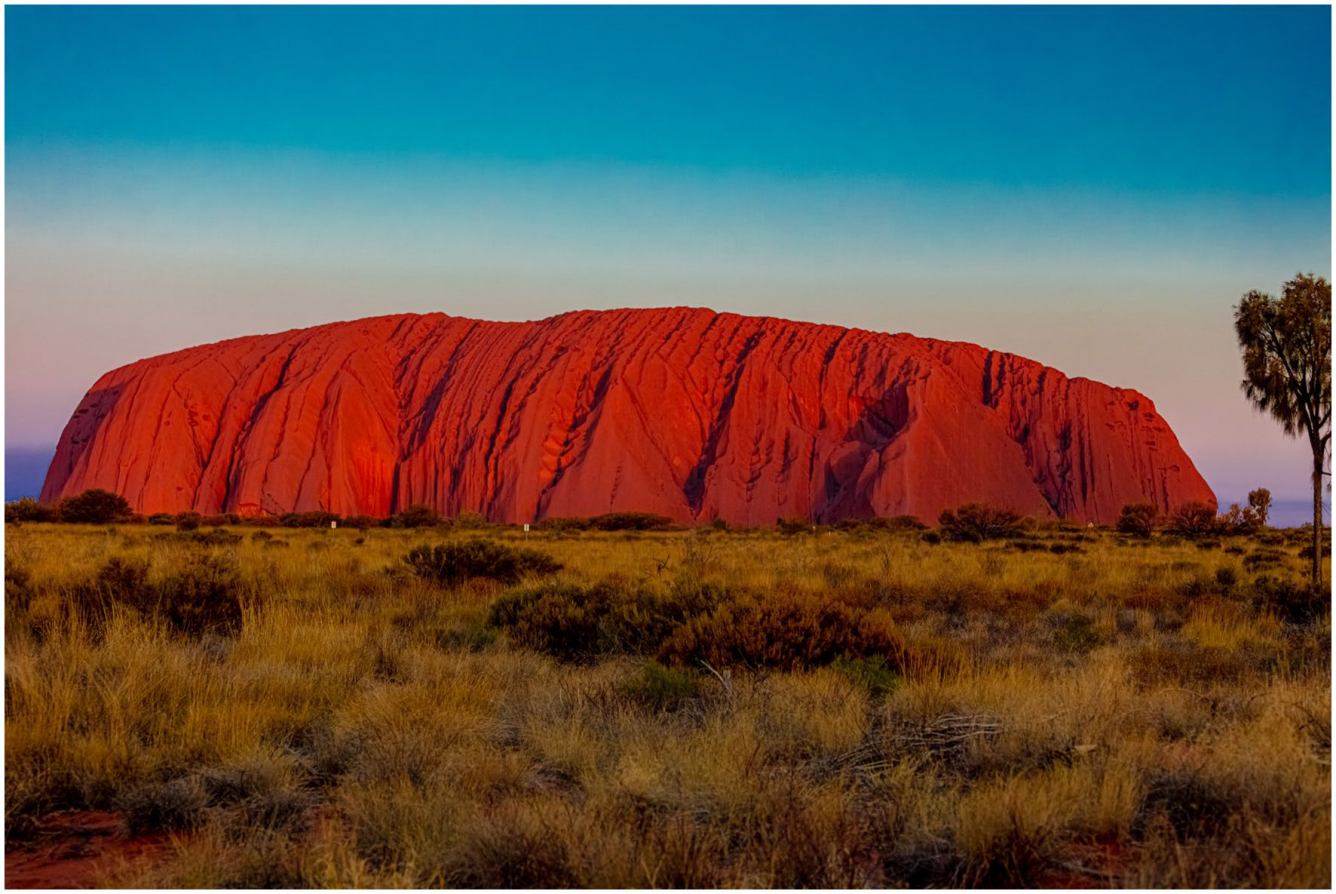
With two articles from Melbourne, Australia in this issue, we highlight one of the iconic side trips you can make from there—Ayers Rock or Uluru—a large sandstone rock formation in the Northern Territory of Australia. In addition to being a sacred site for the Aboriginal population, Uluru has the unique moment at sunset when the reflection of the sun briefly turns it varying shades of red before going dark

- **To the Journals…** Expanding upon my 1980s nostalgia intro, it goes without saying that robotic surgery platforms have transformed a large portion of urologic surgery. Of course there are still strong indications for open surgery, and in some health care systems, it is still preferred for highly skilled laparoscopic surgeons to continue on with minimally invasive procedures without a robot. The costs are certainly a concern and/or barrier, and a topic for another discussion. New robots have the potential to alter the cost equation, bring new instrumentation possibilities and potentially work in even smaller spaces. Since we have essentially had only one manufacturer for so long, many trainees are somewhat sceptical about learning a new robotic platform and would rather just learn the established system and steps. But using my career as an example, it is highly unlikely that any technique one learns in training will stay exactly the same your whole career. Mine started in the 1990s and has run the full gamut of open to laparoscopic to now five different models of Intuitive platforms. Figures 1 and 2 show some slides I pulled from previous lectures to expand on points from the paper by Almujahem and Rha^1^ in this issue—platforms and techniques will change, and new systems bring new value analyses.
- **To the Clinic…** The paper by Anderson et al^2^ is kind of a “bucking the trend” paper on the topic of how to do a radical orchiectomy. I imagine at this point, every urologist alive was taught the technique and rationale for doing a radical orchiectomy—and likely some test questions along the way that made you differentiate when to do or not do a scrotal approach. Is there a downside to the radical/inguinal approach? These authors point out the ilioinguinal nerve damage risk of dissecting in the inguinal canal and illustrate a sub‐inguinal approach that stops at the external inguinal canal. They report oncologic outcomes and follow‐up for 42 cases. Will you try this?
  This group is from Melbourne, Australia, and after a recent visit there, I toured the Outback and visited the famous Ayer's Rock or Uluru as named by the indigenous population (Figure 3). Near the base is an impressive display of solar powered lights—looking like a neural network, Figure 4. So that's my best Australian photo‐analogy—don't damage the light network or it will go dark; and don't damage the ilioinguinal nerve or the patient will complain of pain and/or numbness.
- **To the Drawing Board…** Team science takes significant effort from the principle investigator and a lot of generosity from collaborators to provide patients, data, and editing. Terada et al^3^ report on behalf of the Japanese Urological Oncology Group a 30 institution experience with de novo metastatic prostate cancer. There is a lot of interest in this space with ongoing trials comparing metastatic disease treatments with and without local control, and the recent Stampede trial showing survival benefits in men with low metastatic burden (ref 7 in their paper). These authors confirm longer OS in the radiation group, including higher metastatic burden, and also fewer symptomatic local events. They have a large sample size but it is a retrospective study. I would still keep this in the academic category until more clinical trial evidence is completed—see also an interesting editorial from my colleagues on keeping this question open for now.^4^
- **To the Future…** When I was a fellow, one of my mentors, Christopher Wood, an internationally famous kidney surgeon at MD Anderon Cancer Center, was still early in his career, and had already developed a reputation for being skilled at tackling unusually large kidney tumours. In some cases, other surgeons had attempted but aborted resection and then made the referral. Operating after another surgeon for 20‐plus centimeter tumours is quite an experience for the surgeons, anaesthesiologists, and often the blood bank—a virtual network of bleeding venous plexi surrounding the target. I don't know if he ever wrote up that series, but the catchy title would have been “Resecting the Unresectable.” In a similar fashion, O’Connor et al^5^ from Melbourne, Australia show feasibility of resecting prostate glands with a robot, after a failed open attempt. These are not common, and some of the identified contributors to open failure included obesity, adverse pelvic anatomy, and inguinal hernia repair. This would be a must read if you find yourself in this situation. The authors submitted a video we will post to accompany the text.
